# Editorial: Optical imaging and neurorehabilitation strategies after stroke

**DOI:** 10.3389/fnhum.2022.999725

**Published:** 2022-08-31

**Authors:** Anna-Sophia Wahl, Adam Q. Bauer, Anna Letizia Allegra Mascaro

**Affiliations:** ^1^Brain Research Institute, University of Zurich, Zurich, Switzerland; ^2^Department of Neuroanatomy, Institute of Anatomy, Ludwigs-Maximilians-University, Munich, Germany; ^3^Department of Radiology, Washington University in St. Louis, St. Louis, MO, United States; ^4^Department of Biomedical Engineering, Washington University School of Medicine, St. Louis, MO, United States; ^5^Neuroscience Institute, National Research Council, Pisa, Italy; ^6^European Laboratory for Non-Linear Spectroscopy, University of Florence, Florence, Italy

**Keywords:** ischemia, *in vivo* fluorescence imaging, optogenetics, fNIRS, laser speckle blood flow imaging, optical intrinsic signal imaging, computed tomography perfusion, neurostimulation

The brain elicits a remarkable capacity to respond to and recover from focal injury such as a stroke on different spatial scales. Neuroimaging studies in stroke patients and rodent models show local and global reorganization of functional brain circuits, including shifts in representation of function (see Figure 1). In tandem with these systems-level changes, advances in high-resolution imaging have revealed a plethora of local changes up to the sub-cellular and molecular level. Recent progress in neurophotonics has opened up tremendous opportunities for investigating and manipulating brain circuitry *in vivo*. Optical methods which allow studying cellular and network modifications in real-time can be applied to observe and manipulate network interactions before and after stroke, and also for developing rehabilitative strategies designed to improve recovery of function. This Research Topic presents some of the latest applications of neurophotonics in preclinical and clinical stroke research. Contributions ranged from state-of-the-art neuroimaging, different neuromodulatory strategies combined with light-based monitoring as well as the assessment of different treatment approaches using optical methods in preclinical stroke models and clinical trials.

**Figure 1:**
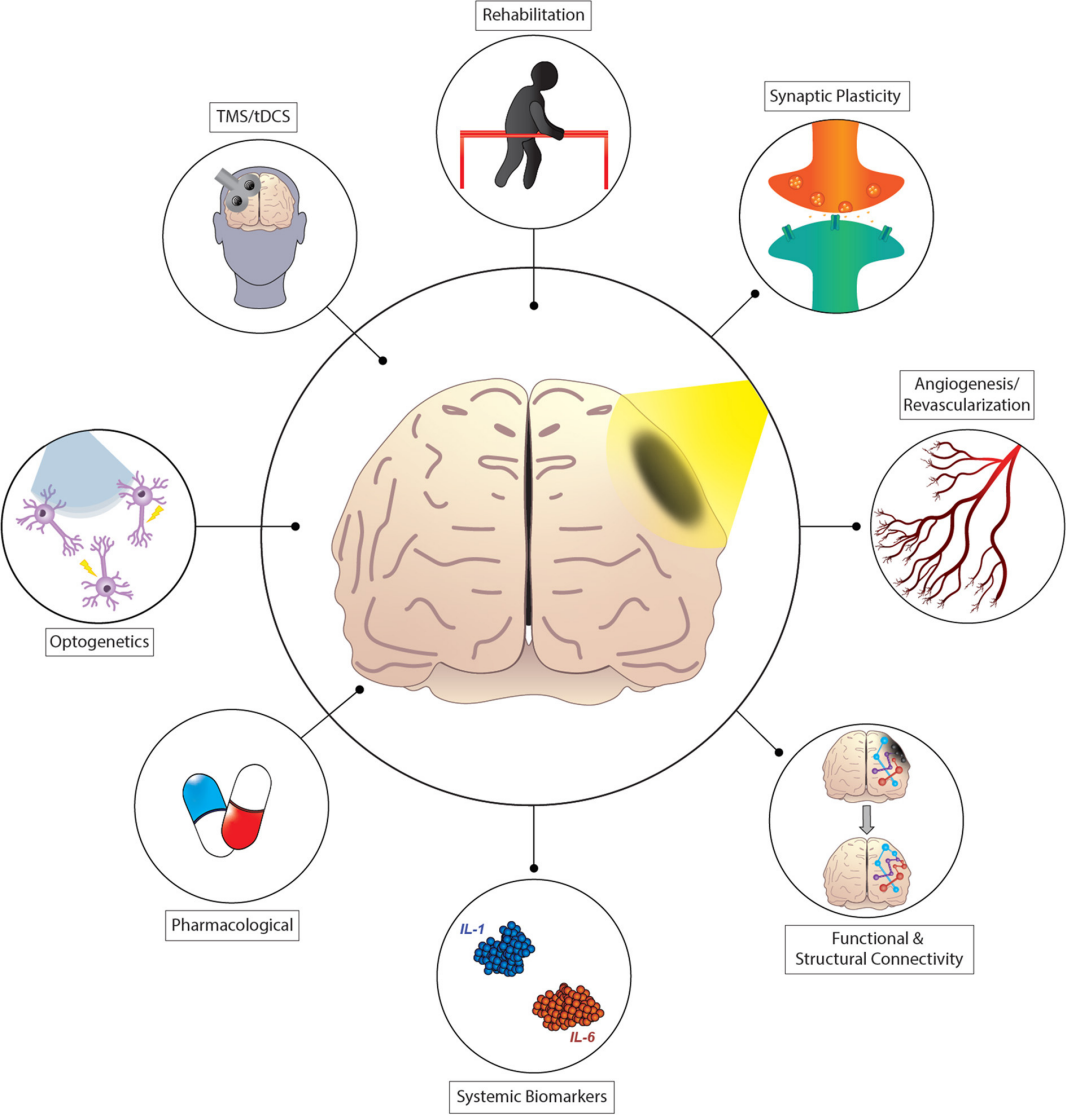
Shining light on recovery after stroke. Recovery from stroke involves the formation of new or alternative structural and functional connections. These processes are facilitated by changes in the expression of genes affecting neuronal plasticity, angiogenesis, and other mechanisms. Interventional strategies designed to affect recovery processes include physical therapy, pharmacological treatment, or brain stimulation techniques such as transcranial magnetic stimulation, direct current stimulation (TMS/tDCS), and optogenetic targeting strategies.

Gilmore et al. present a novel application of neurophotonics techniques to investigate brain functionality after stroke. In this study, functional near-infrared spectroscopy (fNIRS) is used to capture brain activation during language and cognitive tasks. The authors compared healthy controls to individuals with left hemisphere stroke-induced aphasia. Healthy subjects activated core language regions such as the left inferior frontal gyrus pars opercularis and pars triangularis as well as the bilateral middle frontal gyri as expected. However, in stroke patients, regions outside of these traditional networks were identified. Among these areas were the left superior frontal and supramarginal gyrus as well as the right precentral gyrus. This study may serve as a reference when interpreting treatment-related changes in brain activation patterns using fNIRS in stroke patients with aphasia.

Two reviews highlighted the importance of neuromodulation and rehabilitation therapies on behavioral recovery and cortical plasticity. In the review by Storch et al. light-based imaging and manipulation techniques (optogenetics) are discussed that are currently used in the field of stroke and neuromodulatory intervention. Interestingly, Storch et al. propose a roadmap for implementing the next generation of strategies to promote functional recovery after stroke. In the review by Wen et al. repetitive transcranial magnetic stimulation (rTMS) applied to the mylohyoid cortical region was evaluated as a possible treatment of post-stroke dysphagia. In their meta-analysis, the authors included 11 randomized clinical trials with 463 dysphagia patients. Results suggest that rTMS is particularly beneficial for treating post-stroke dysphagia when used in combination with traditional swallowing exercises. The authors also report that the stimulation frequency is crucial for the final outcome parameters. High-frequency stimulation with rTMS had a greater effect size than low-frequency stimulation. However, whether the stroke affected or unaffected hemisphere was stimulated did not significantly influence recovery.

Four studies showcase the practicality of specific optical methods in preclinical and clinical trials for assessing different novel treatment options for stroke. In the study by Wang et al. the authors combined laser speckle contrast imaging and optical intrinsic signal imaging to investigate the neuroprotective role of AUDA, an anti-inflammatory drug, in rats following photothrombosis. Acute administration (<24 h post stroke) of AUDA reduced the size of the ischemic penumbra and prevented the extension of the ischemic core. To explain these findings, functional imaging at 3 days after stroke revealed AUDA restored cerebral blood flow and local oxygen metabolism, while structural imaging revealed AUDA-associated increases in angiogenesis compared to controls. At the cellular level, immunofluorescence staining indicated astrocytes within the penumbral region may play an important role in protecting neurons from apoptotic injury. AUDA thus presents a novel therapy for potentially increasing long-term neuroprotection following focal ischemia.

In the study by Wei et al. a different form of imaging (computed tomography perfusion, CTP) was applied to test hyperbaric oxygen treatment in patients with hypertensive intracerebral hemorrhage. The team provides evidence suggesting a beneficial role for hyperbaric oxygen treatment following the standard treatment of puncture and drainage. Specifically, hyperbaric oxygen treatment improved cerebral perfusion parameters and was also associated with better neurological outcome. Although this study is still very experimental, it provides a first hint for the practicability and efficiency of hyperbaric oxygen therapy demanding further research into the underlying neurobiological mechanisms.

Scaglione et al. in their paper tested the idea of whether neural activity in response to a motor output can be used to assess the effect of therapeutic interventions after stroke. They demonstrated that task-evoked cortical activation sequences obtained from wide-field calcium imaging can be used as an indicator of disease state and allowed tracking recovery without selecting any specific feature of the cortical neuronal responses. The authors propose a euclidean classifier strategy applied to a variety of signals to maximize the development and assessment of many rehabilitative approaches. The classification method described in this study could be implemented using other neural signals such as EEG, fMRI, or FNIRs, and could be used for both the development and assessment of different therapeutic strategies.

Finally, Zhang et al. used MRI detection in a transient middle cerebral artery occlusion model (tMCAO) in rats to assess the influence of different forms of enriched rehabilitation on functional recovery. While it has been previously demonstrated that environmental enrichment promotes functional recovery after stroke, it is not understood which types of enrichment provide the greatest benefit. To address this gap, the authors examined 4 different types of housing conditions on recovery: physical enrichment, social enrichment, combined physical, and social enrichment, and standard housing. Exposure to the combination of both physical and social enrichment yielded the greatest beneficial effect on neurological recovery and was also associated with the greatest increases in microvessel density within the penumbra and expression levels of genes associated with angiogenesis. This study highlights the importance of combined multisensory and social stimulation paradigms after injury, and might provide guidance for interventional strategies designed to accelerate processes important for recovery after stroke.

In summary, this Research Topic demonstrates the impressive range of optical imaging technologies in stroke research. Contributions spanned from structural imaging to multimodal functional imaging and novel neuromodulatory interventions in preclinical and clinical settings. The ongoing development of neurophotonics approaches will increase the capability of understanding the pathophysiological progression of ischemia and mechanisms important for functional recovery. While future work is still needed, multidisciplinary, collaborative efforts aligning pre-clinical and clinical research offer the highest probability for the development and improvement of stroke treatments.

## Author contributions

All authors listed have made a substantial, direct, and intellectual contribution to the work and approved it for publication.

## Funding

This research has been supported by the Branco Weiss Society in Science Fellowship, Swiss National Science Foundation (No. 192678), Hurka Foundation, Synapsis Foundation, Wrangell Fellowship (A-SW); by the National Institute of Health grants R01-NS102870 and K25-NS083754, and the McDonnell Center for Systems Neuroscience (AB); by the Regione Toscana-Bando Ricerca Salute 2018, Grant Number 20RSVP, project NIMBLE and by the FCRF, SIME 2018/1179 id#24055 for the project STROKELAB2BED (AA).

## Conflict of interest

The authors declare that the research was conducted in the absence of any commercial or financial relationships that could be construed as a potential conflict of interest.

## Publisher's note

All claims expressed in this article are solely those of the authors and do not necessarily represent those of their affiliated organizations, or those of the publisher, the editors and the reviewers. Any product that may be evaluated in this article, or claim that may be made by its manufacturer, is not guaranteed or endorsed by the publisher.

